# Preoperative assessment for the risk of Clavien-Dindo classification ≥ II grade complications after total parathyroidectomy without auto-transplantation in patients with secondary hyperparathyroidism

**DOI:** 10.3389/fendo.2025.1705798

**Published:** 2025-12-10

**Authors:** Yuanzhi Ni, Lindi Xu, Zhenli Li, Liang Li, Shihang Yan, Guangming Cheng, Chunhui Wang, Yufu Tang

**Affiliations:** 1 Department of Hepatobiliary and Thyroid Surgery, General Hospital of Northern Theater Command, Shenyang, China; 2 China Medical University, Shenyang, China; 3 Dalian Medical University, Dalian, China; 4 Department of General Surgery, the 963rd Hospital of the Joint Service Support Force, Jiamusi, China

**Keywords:** secondary hyperparathyroidism, total parathyroidectomy, Clavien-Dindo grade, nomogram, complications

## Abstract

**Objective:**

Complications following total parathyroidectomy (TPTX) without autotransplantation adversely affect the prognosis of patients with secondary hyperparathyroidism (SHPT). This study aimed to identify preoperative risk factors of Clavien-Dindo grade ≥ II complications after TPTX in SHPT patients, and develop a visualized model to predict the risk of complications (≥ grade II).

**Methods:**

Clinical data were retrospectively collected from SHPT patients who underwent TPTX at our center between January 2013 and March 2025. The cohort was chronologically divided into a training set (n=552) and a validation set (n=236) in a 7:3 ratio. Postoperative complication severity was assessed using the Clavien-Dindo classification system. Patients were stratified into two groups based on complication grade: a high-risk group (grade ≥II) and a low-risk group (grade <II or no complications). Independent risk factors of Clavien-Dindo grade ≥ II complications were identified through univariate and multivariate logistic regression analyses. A nomogram was developed using these independent risk factors. The performance of this model was then evaluated using the validation set.

**Results:**

Among 788 patients, 546 (69.3%) developed postoperative complications, including 369 cases (46.8%) of grade ≥ II complications. Multivariate analysis identified serum alkaline phosphatase, intact parathyroid hormone, serum calcium, and serum potassium as independent risk factors of grade ≥II complications. The nomogram achieved good concordance indexes of 0.826 (95% confidence interval (CI), 0.778–0.874) and 0.807 (95% confidence interval (CI), 0.739–0.874) in predicting the risk of postoperative complications (grade ≥ II) in the training and validation cohorts, respectively, with well-fitted calibration curves. Both the decision curve and the clinical impact curve indicated substantial clinical benefit provided by the nomogram.

**Conclusion:**

A nomogram was developed and validated to predict complications (grade ≥ II) following TPTX without autotransplantation in patients with SHPT. This model may aid in identifying high-risk patients and informing perioperative decision-making.

## 1 Introduction

Secondary hyperparathyroidism (SHPT) is a common complication of chronic kidney disease (CKD), with an incidence exceeding 50% in CKD patients and reaching 90% in patients with end-stage renal disease (ESRD) (1). As the core manifestation of the chronic kidney disease-mineral and bone disorder (CKD-MBD) syndrome, SHPT is closely associated with multisystem damage, including increased risks of cardiovascular calcification, fractures, and elevated all-cause mortality (2–4). Therefore, positive control of SHPT is crucial for improving the prognosis of CKD patients. For patients with refractory SHPT unresponsive to medical therapy, parathyroidectomy (PTX) has been proven as a long-term effective treatment (5). This procedure significantly reduces serum intact parathyroid hormone (iPTH) levels and alleviates clinical symptoms such as bone pain and pruritus (6, 7). With the widespread application of intraoperative adjunct techniques (e.g., intraoperative PTH monitoring, nerve monitoring systems), the surgical success rate of PTX has been markedly improved (8). However, the high risk of postoperative complications remains as an unsolved concern. Within 30 days after PTX, the readmission rate due to postoperative complications reaches 17.2% (9), including infection-related complications, electrolyte imbalances, nerve injuries, and cardiovascular complications. Early precaution and management of high-risk population of postoperative complications may reduce the incidence of severe complications and improve prognostic outcomes. Current evidence predominantly centers on risk factors and prediction models for electrolyte imbalances such as hypocalcemia, hungry bone syndrome (HBS) and hyperkalemia, while research on other complications remains insufficient. Moreover, the comprehensive studies defining the association between clinical factors and the severity of complications are lacking.

This study aims to explore postoperative complications after TPTX without auto-transplantation in SHPT patients based on Clavien-Dindo classification system. Especially, we identified independent risk factors for patients with ≥ grade II complications, who warranted systematic treatments or interventions. Using these significant predictors, a nomogram was further developed and validated, which aids as a preoperative risk assessment tool in identifying high-risk patients and facilitating intervention strategies.

## 2 Materials and methods

### 2.1 Patients

This study collected clinical and pathological data of consecutive SHPT patients who underwent TPTX without auto-transplantation at the General Hospital of Northern Theater Command from January 2013 to March 2025. The study protocol was approved by the Ethics Committee of the General Hospital of Northern Theater Command (Approval No. Y(2025)177). All patients were informed of surgical risks and procedures and provided written informed consent.

### 2.2 Eligibility criteria

The inclusion criteria were as follows: (1) Patients requiring PTX based on the Kidney Disease Outcomes Quality Initiative (KDOQI) guidelines, including persistent elevation of serum intact parathyroid hormone (iPTH) >800 pg/mL, uncontrolled hypercalcemia with hyperphosphatemia, severe clinical symptoms (e.g., bone/joint pain, muscle weakness, or refractory pruritus), or failure of medical therapy; (2) Patients undergoing TPTX without autotransplantation; (3) Patients with successful surgery had intact parathyroid hormone (iPTH) levels <60 pg/mL on postoperative day 1 (POD 1).Exclusion criteriawere as follows:(1) Patients with surgical failure;(2) Patients with incomplete clinical data;(3) Patients undergoing secondary surgery due to recurrent SHPT.Successful TPTX is defined as: the core principle of resecting all intraoperatively identifiable parathyroid glands; if all glands cannot be fully exposed due to anatomical variations, ectopy, or exploration difficulties, at least ≥3 glands must be resected; if the patient has only 3 or fewer parathyroid glands due to congenital variations, all identifiable glands must be completely resected. All the above situations must be combined with an iPTH level <60 pg/mL within 24 hours postoperatively as a necessary condition for determining success (10). Patients who underwent surgery between January 2013 and August 2022 were assigned to the training set for the development of the prediction model, and those who underwent surgery after that were assigned to the validation set to evaluate the accuracy of the model.

### 2.3 Perioperative management and surgical procedures

Routine preoperative examination included laboratory tests (serum iPTH, serum alkaline phosphatase, serum calcium, serum phosphate, serum potassium, hemoglobin, albumin, serum creatinine, urea, prothrombin time, and fibrinogen) and ultrasonography of the thyroid and parathyroid glands. All patients had dialysis within 24 h before surgery. Preoperative diagnosis followed the clinical practice guidelines of Kidney Disease: Improving Global Outcomes (KDIGO) (3, 11). All surgeries were performed by Dr. Guangming Cheng and his surgical team.

### 2.4 Classification of postoperative complications

This study classified postoperative complication severity using the Clavien-Dindo classification system (12), The Clavien-Dindo classification system categorizes postoperative complications into grades I to V based on the severity of the complications and the requirement for corresponding treatment. Grade I: Abnormal changes occurring after surgery that do not require medication, surgery, endoscopy, or radiotherapy, but include complications requiring antiemetics, antipyretics, analgesics, electrolytes, and physical therapy, as well as wound infections requiring open drainage at the bedside. Grade II: Requires drug therapy other than those used in Grade I, including blood transfusion and total parenteral nutrition. Grade III: Requires intervention measures such as surgery, endoscopy, or radiotherapy. Grade IV: Life-threatening complications, including central nervous system complications and those requiring intensive care or management in the intensive care unit. Grade V: Death. Based on whether the patients required necessary systematic treatment or intervention, we divided them into two groups: a high-risk group (Clavien-Dindo ≥ grade II complications) and a low-risk group (Clavien-Dindo < grade II complications or no complications). Postoperative complications included surgery-related issues occurring within 30 days postoperatively, diagnosed through clinical symptoms, laboratory tests, and imaging studies documented in medical records. These complications included mild hypocalcemia, severe hypocalcemia, mild to moderate hyperkalemia, severe hyperkalemia, superior laryngeal nerve injury, recurrent laryngeal nerve injury, arrhythmia, heart failure, hemorrhage, incision infection, pulmonary infection, arteriovenous fistula occlusion, and even death. Postoperative serum calcium < 2.20 mmol/L was defined as hypocalcemia, while serum calcium < 1.80 mmol/L was defined as severe hypocalcemia. Postoperative serum potassium ≥ 5.5 mmol/L was defined as hyperkalemia. Severe hyperkalemia was defined as serum potassium ≥ 6.5 mmol/L or serum potassium > 6.0 mmol/L accompanied by electrocardiographic changes (13).

Notably, the Clavien-Dindo system grades complications based on the level of intervention required to manage them. Consequently, the same type of complication might be assigned different grades depending on the intensity of intervention needed. For example, postoperative hyperkalemia managed without specific intervention is grade I; hyperkalemia requiring pharmacotherapy for control is grade II; severe hyperkalemia necessitating emergency dialysis (distinct from the patient’s scheduled routine dialysis) constitutes a grade III complication (14).

### 2.5 Statistical analysis

Data analysis was performed using SPSS Statistics (version 27.0) and R software (version 4.4.3). Continuous variables are presented as mean ± standard deviation or median with interquartile range (IQR), based on data distribution. Categorical variables are expressed as frequencies and percentages. Group comparisons employed: Student’s t test or Mann-Whitney U test for continuous variables, and chi-squared test or Fisher’s exact test for categorical variables, as appropriate. Univariable logistic analysis was used to identify clinically relevant variables associated with the postoperative occurrence of ≥ grade II complications in the training cohort. All variables associated with the occurrence of ≥ grade II complications at a significant level were considered as candidate variables for multivariate logistic analysis. Based on the results of multivariate logistic regression analysis, a nomogram was constructed using R software. The predictive performance of the nomogram was measured with the concordance index (C index) and calibration with 1000 bootstrap samples to decrease the overfit bias. P value of less than 0.05 was considered to be statistically significant.

## 3 Results

### 3.1 Patient characteristics

A total of 788 patients who met the inclusion and exclusion criteria were enrolled, with their clinical characteristics summarized in **Table 1**. Postoperative complications occurred in 546 patients (69.3%), presenting as mild hypocalcemia, severe hypocalcemia, mild to moderate hyperkalemia, severe hyperkalemia, superior laryngeal nerve injury, recurrent laryngeal nerve injury, arrhythmia, heart failure, hemorrhage, incision infection, pulmonary infection, arteriovenous fistula occlusion, or death (**Table 2**). Patients might develop one or multiple complications, with the final complication count determined by the most severe type observed. Among these cases, 369 patients (46.8%) experienced ≥ grade II complications. Notably, 4 patients died due to respiratory failure secondary to pulmonary infection, acute myocardial infarction, heart failure, or metabolic acidosis. The remaining patients were discharged after recovery and received subsequent symptomatic treatment. The cohort was chronologically divided into a training set (n=552) and a validation set (n=236) in a 7:3 ratio. This approach ensures the consistency of data distribution and allows better detection of the model’s predictive ability by training with historical data and testing with future data. No significant differences were noted in clinical characteristics between the training and validation sets (**Table 3**). The incidence of ≥ grade II complications was 46.5% in the training set and 47.4% in the validation set, respectively.

**Table 1:** Clinical characteristics of 788 patients undergoing TPTX.

Variable	All patients (n=788)
Gender, %	
Male	438 (55.6%)
Female	350 (44.4%)
Initial kidney disease, %	
Nephritis	175 (22.2%)
Hypertensive nephropathy	93 (11.8%)
Diabetic nephropathy	26 (3.3%)
Polycystic kidney	20 (2.5%)
Others	121 (15.3%)
Unknow	353 (44.8%)
iPTH (pg/mL), %	
≤1000	123 (15.6%)
>1000	665 (84.4%)
Alkaline phosphatase (U/L), %	
≤420	565(71.7%)
>420	223(28.3%)
Age, median (IQR), y	47 (38.0-56.0)
BMI, median (IQR), kg/m2	22.3 (20.0-24.8)
Duration of dialysis, median (IQR), y	8.0 (6.0-10.0)
Hemoglobin, mean (SD), g/L	107.26 (17.9)
Albumin, median (IQR), g/L	38.2 (35.7-40.8)
Blood urea nitrogen, median (IQR), mmol/L	21.0 (16.7-26.2)
Serum creatinine, median (IQR), mmol/L	930.4 (758.1-1150.6)
Prothrombin time, median (IQR), s	13.4 (12.8-13.9)
Fibrinogen, median (IQR), g/L	4.3 (3.6-5.0)
Serum calcium, median (IQR), mmol/L	2.5 (2.3-2.6)
Serum potassium, median (IQR), mmol/L	4.7 (4.2-5.1)
Serum phosphate, median (IQR), mmol/L	2.4 (2.0-2.8)

BMI, body mass index; iPTH, intact parathyroid hormone; IQR, interquartile range; SD, standard deviation.

**Table 2:** Postoperative complications of 788 patients undergoing TPTX.

Complications	Grade I	Grade II	Grade III	Grade IV	Grade V
Mild Hypocalcemia	89	60	0	0	0
Severe Hypocalcemia	27	198	0	0	0
Mild to Moderate Hyperkalemia	102	88	1	0	0
Severe Hyperkalemia	0	21	48	0	0
Superior laryngeal nerve injury	0	2	0	0	0
Recurrent laryngeal nerve injury	0	22	0	0	0
Arrhythmia	0	6	0	0	0
Heart failure	0	6	0	0	0
Hemorrhage	6	0	1	0	0
Incision infection	2	2	0	0	0
Pulmonary infection	0	7	0	0	0
Arteriovenous fistula occlusion	0	12	0	0	0
Death	0	0	0	0	4

**Table 3:** Clinical characteristics of patients in the training and validation sets.

Variable	Training(n=552)	Validation(n=236)	P value
Gender			0.775
Male	305 (55.3)	133 (56.4)	
Female	247 (44.7)	103 (43.6)	
Dialysis modalities			0.990
Hemodialysis	521 (94.6)	222 (94.5)	
Peritoneal dialysis	30 (5.4)	13 (5.5)	
iPTH, pg/ml			0.187
≤1000	80 (14.5)	43 (18.2)	
>1000	472 (85.5)	193 (81.8)	
Alkaline phosphatase, U/L			0.985
≤420	398 (72.1)	170 (72.0)	
>420	154 (27.9)	66 (28.0)	
Hemoglobin, mean (SD), g/L	107 (17.82)	107 (18.30)	0.744
Age, median (IQR), y	47.0 (38.0-56.0)	46.0 (37.0-54.0)	0.212
BMI, median(IQR), kg/m2	22.29 (20.04-24.93)	22.48 (20.02-24.68)	0.516
Duration of kidney disease, median(IQR), y	10 (7-13)	10 (7-13)	0.742
Duration of dialysis, median(IQR), y	8 (6-10)	8 (6-10)	0.871
Albumin, median (IQR), g/L	38.2 (35.8-40.6)	38.3 (35.5-41.4)	0.329
Blood urea nitrogen, median(IQR), mmol/L	20.6 (16.5-26.0)	21.9 (16.8-26.9)	0.195
Serum creatinine, median (IQR), mmol/L	928 (749-1142)	950 (765-1172)	0.391
Prothrombin time, median (IQR), s	13.4 (12.8-14.0)	13.3 (12.7-13.9)	0.356
Fibrinogen, median (IQR), g/L	4.37 (3.64-5.02)	4.22 (3.63-4.96)	0.402
Serum calcium, median (IQR), mmol/L	2.45 (2.31-2.57)	2.49 (2.31-2.60)	0.196
Serum phosphate, median (IQR), mmol/L	2.37 (2.03-2.78)	2.37 (2.04-2.74)	0.995
Serum potassium, median (IQR), mmol/L	4.69 (4.20-5.14)	4.67 (4.25-5.10)	0.804

BMI, body mass index; iPTH, intact parathyroid hormone; IQR, interquartile range-;SD, standard deviation.

### 3.2 Univariate and multivariate logistic-regression analyses in the training set

Univariate logistic regression analysis identified potential factors associated with ≥ grade II complications. All variables analyzed were derived from preoperative data. Univariate analysis demonstrated that age, serum alkaline phosphatase (ALP), fibrinogen, serum iPTH, preoperative serum calcium, serum phosphate, and serum potassium were significantly associated with the occurrence of ≥ grade II complications (*P* < 0.05) (**Table 4**). These significant parameters were further analyzed upon multivariate logistic regression. The results revealed serum alkaline phosphatase [OR (95% CI) = 0.319(0.179-0.567), *P* < 0.001], intact parathyroid hormone [OR (95% CI) = 0.474(0.271-0.828), *P=*0.009], serum calcium [OR (95% CI) = 0.062 (0.022-0.178), *P* < 0.001], and serum potassium [OR (95% CI) = 2.500 (1.772-3.527), *P* < 0.001] as independent risk factors of ≥ grade II complications (**Table 5**).

**Table 4:** Univariate analysis in the training set.

Variable	OR(95%CI)	P value
Gender	1.082 (0.751-1.558)	0.672
Dialysis modalities	0.924 (0.431-1.982)	0.840
Diabetes	1.043 (1.013-1.073)	0.472
Hemoglobin, mean (SD), g/L	0.992 (0.982-1.002)	0.127
Age, median (IQR), y	0.974 (0.959-.990)	0.002
BMI, median (IQR), kg/m2	1.009 (0.964-1.057)	0.691
Duration of kidney disease, median(IQR), y	1.014 (0.977-1.052)	0.479
Duration of dialysis, median (IQR), y	0.971 (0.923-1.022)	0.260
Alkaline phosphatase, ≤420vs>420, U/L	0.299 (0.184-0.487)	<0.001
Albumin, median (IQR), g/L	1.006 (0.987-1.026)	0.538
Blood urea nitrogen, median (IQR), mmol/L	1.022 (0.994-1.051)	0.129
Serum creatinine, median (IQR), mmol/L	1.000 (1.000-1.001)	0.405
Prothrombin time, median (IQR), s	1.087 (0.894-1.320)	0.403
Fibrinogen, median (IQR), g/L	1.415 (1.180-1.697)	<0.001
iPTH, ≤1000 vs >1000, pg/ml	0.307 (0.189-0.498)	<0.001
Serum calcium, median (IQR), mmol/L	0.085 (0.033-0.219)	<0.001
Serum phosphate, median (IQR), mmol/L	1.685 (1.210-2.348)	0.002
Serum potassium, median (IQR), mmol/L	2.465 (1.814-3.348)	<0.001

BMI, body mass index; iPTH, intact parathyroid hormone; OR, odds ratio; CI, confidence interval.

**Table 5:** Multivariate analysis in the training set.

Variable	β	OR(95%CI)	P value
Age, median (IQR), y	-0.012	0.988 (0.969-1.007)	0.228
Alkaline phosphatase, ≤420vs>420, U/L	-1.143	0.319 (0.179-0.567)	<0.001
Fibrinogen, median (IQR), g/L	0.181	1.199 (0.976-1.473)	0.084
iPTH, ≤1000 vs >1000, pg/ml	-0.747	0.474 (0.271-0.828)	0.009
Serum calcium, median (IQR), mmol/L	-2.777	0.062 (0.022-0.178)	<0.001
Serum phosphate, median (IQR), mmol/L	0.450	1.568 (1.023-2.402)	0.059
Serum potassium, median (IQR), mmol/L	0.916	2.500 (1.772-3.527)	<0.001

iPTH, intact parathyroid hormone; OR, odds ratio; CI, confidence interval.

### 3.3 Development and validation of the prediction model

**Figure 1:**
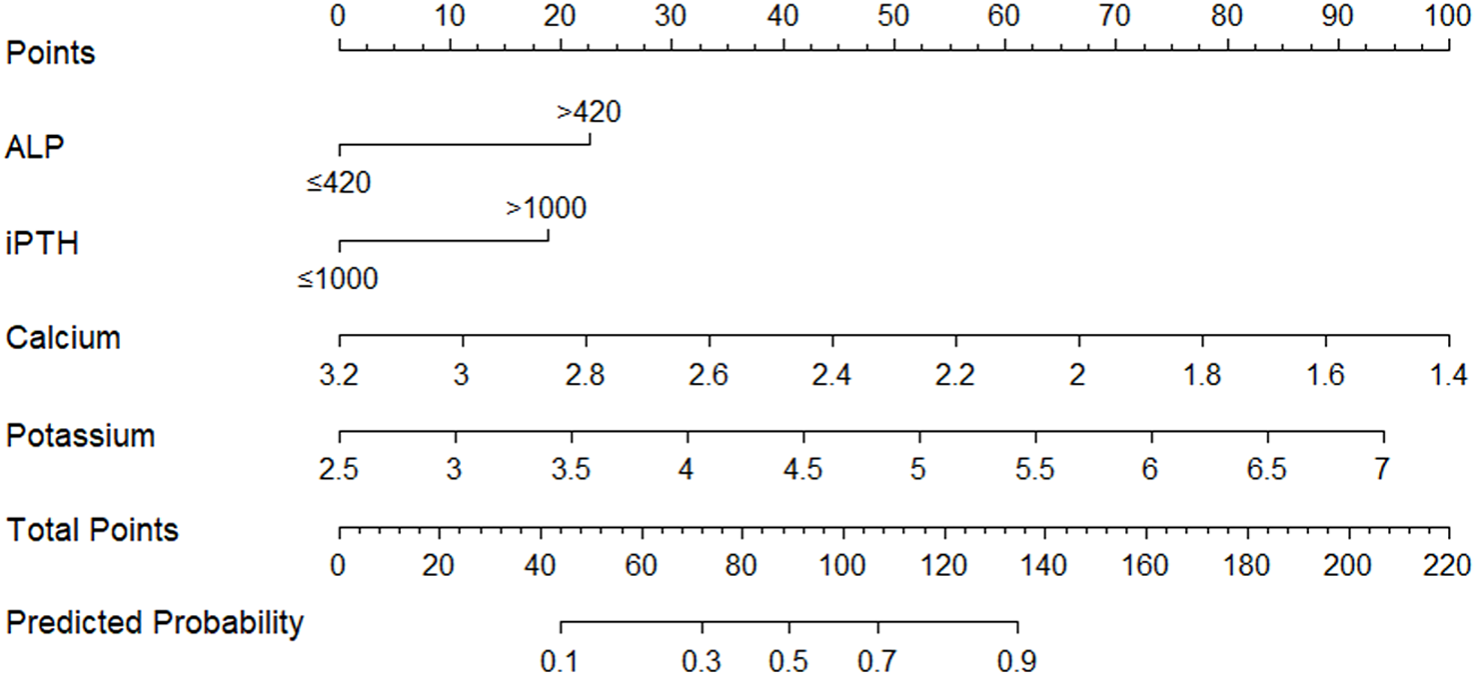
Nomogram for preoperative prediction of Clavien-Dindo grade ≥II complications following total parathyroidectomy (TPTX) without auto-transplantation in patients with secondary hyperparathyroidism (SHPT).

A nomogram incorporating 4 independent risk factors, ALP, iPTH, serum calcium, and serum potassium, was developed to predict the risk of ≥ grade II complications after TPTX in SHPT patients. The model assigned scores to each factor via the “Points” axis, summed the total score, and predicted the approximate probability of complications based on the total score. The total score demonstrated a positive correlation with complication probability. Through ROC curve analysis combined with Youden index calculation, we determined the optimal cut-off value as 106.1 points, and based on this, patients were clearly divided into the high-risk group (>106.1 points) and low-risk group (<106.1 points) for developing overall complications of Clavien-Dindo grade ≥II after surgery(**Figure 1**).

**Figure 2:**
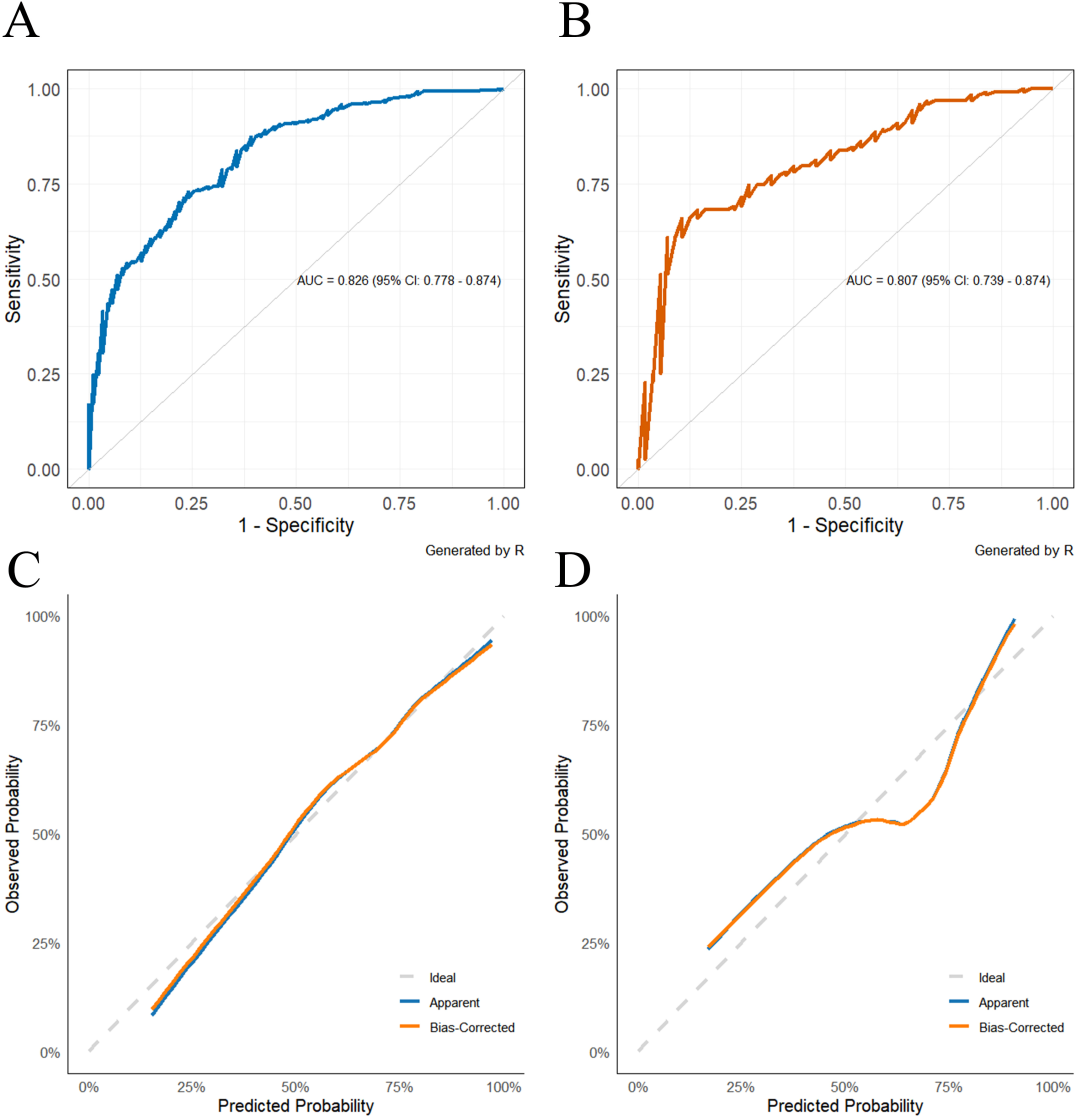
**(A)** Receiver operating characteristics (ROC) curve of the model in the training cohort. X-axis: 1 - Specificity (False Positive Rate); Y-axis: Sensitivity (True Positive Rate). **(B)** Receiver operating characteristics (ROC) curve of the model in the validation cohort. X-axis: 1 - Specificity (False Positive Rate); Y-axis: Sensitivity (True Positive Rate). **(C)** Calibration curve in training cohort. X-axis: Model-predicted event occurrence probability; Y-axis: Actually observed event occurrence probability. **(D)** Calibration curve in validation cohort. X-axis: Model-predicted event occurrence probability; Y-axis: Actually observed event occurrence probability.

The discriminatory performance of the nomogram was evaluated using receiver operating characteristic (ROC) curve analysis. Results showed area under the curve (AUC) values of 0.826 (95% CI: 0.778-0.874) for the training set and 0.807 (95% CI: 0.739-0.874) for the validation set (**Figures 2A, B**). Sensitivity and specificity were 74.6% and 86.5% in the training set, and 78.6% and 81.8% in the validation set, respectively, indicating strong discriminatory ability (**Table 6**). Calibration curve analysis revealed that predicted probabilities were of high agreement with the ideal reference line (45-degree diagonal), suggesting excellent calibration performance (**Figures 2C, D**). The Hosmer-Lemeshow test further validated model fit quality, with no statistically significant differences in the training set (χ² = 7.088, *P* = 0.527) or validation set (χ² = 9.495, *P* = 0.302), exhibiting favorable model fit.

**Table 6:** Accuracy of the prediction model for Clavien-Dindo grade ≥II complications after TPTX.

Variable	Value (95%CI)	
	Training	Validation
Area under ROC curve	0.826(0.778-0.874)	0.807(0.739-0.874)
Sensitivity, %	0.746(0.627-0.837)	0.786(0.605-0.898)
Specificity, %	0.865(0.720-0.941)	0.818(0.615-0.927)
Positive predictive value, %	0.904(0.794-0.958)	0.846(0.665-0.938)
Negative predictive value, %	0.667(0.525-0.783)	0.750(0.551-0.880)
Positive likelihood ratio	5.521(2.612-14.400)	4.321(1.923-12.726)
Negative likelihood ratio	0.294(0.173-0.438)	0.262(0.107-0.500)

**Figure 3:**
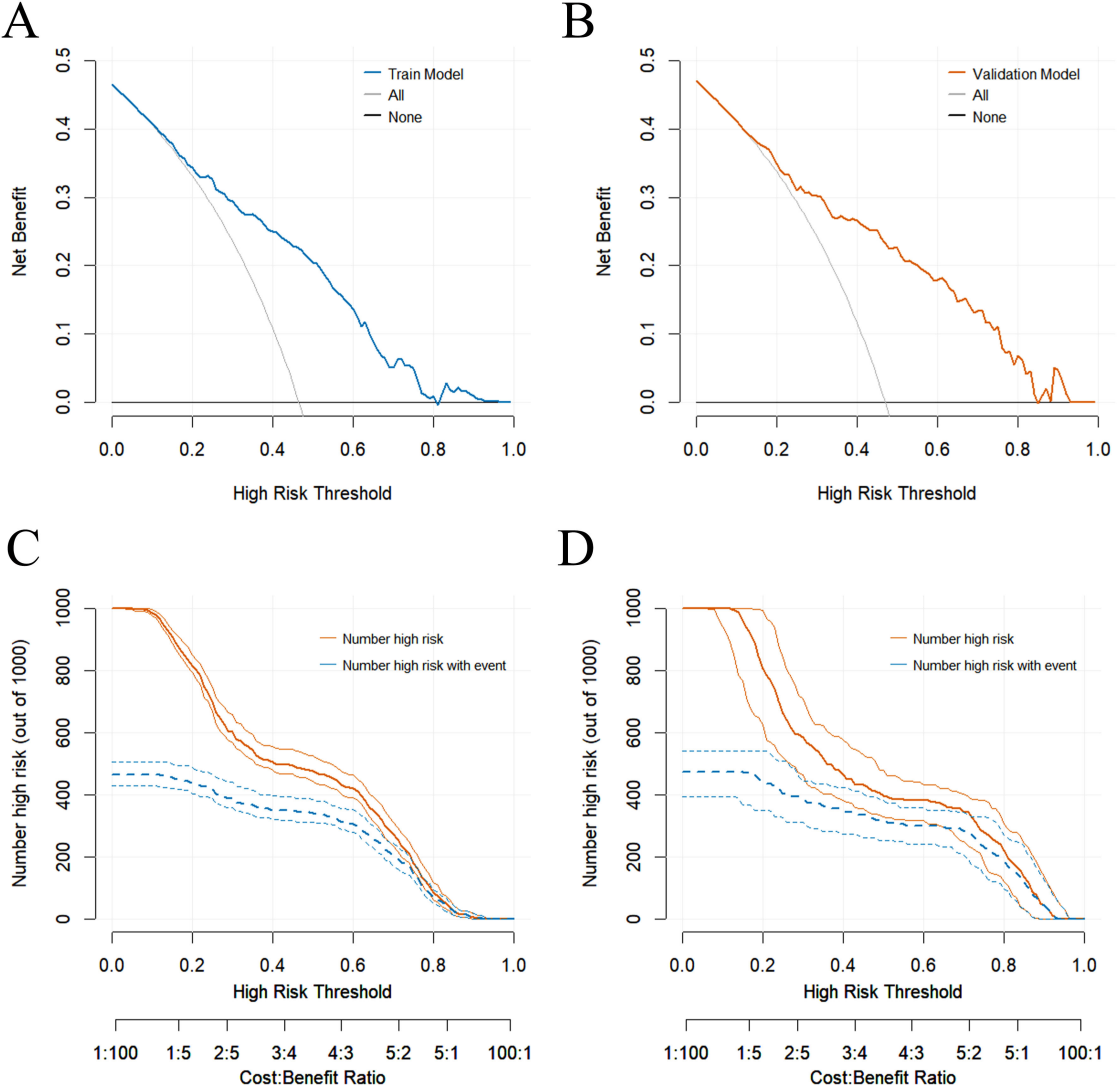
**(A)** Decision curve analysis of the model in the training cohort. X-axis: Threshold probability (minimum acceptable probability of event occurrence); Y-axis: Net benefit (actual clinical value). **(B)** Decision curve analysis of the model in the validation cohort. X-axis: Threshold probability (minimum acceptable probability of event occurrence); Y-axis: Net benefit (actual clinical value). **(C)** Clinical impact curve of the model in the training cohort. X-axis: Threshold probability (minimum acceptable probability of event occurrence); Y-axis: Number of individuals predicted to be high-risk by the model per 1000 participants. **(D)** Clinical impact curve of the model in the validation cohort. X-axis: Threshold probability (minimum acceptable probability of event occurrence); Y-axis: Number of individuals predicted to be high-risk by the model per 1000 participants.

Decision curve analysis (DCA) demonstrated significant net benefit advantages within threshold probability ranges of 25%–92% for the training set and 25%–90% for the validation set (**Figures 3A, B**). Clinical impact curve (CIC) indicated that when the threshold probability was high, the number of high-risk subjects (nomogram-predicted positive cases) closely matched the number of true high-risk outcome subjects (true positive cases) (**Figures 3C, D**), robustly validating the nomogram’s clinical applicability. Collectively, these analyses confirmed the model’s reliable predictive performance and diagnostic accuracy.

## 4 Discussion

This study identified 4 independent predictors associated with an increased risk of Clavien-Dindo ≥ II grade complications after TPTX in SHPT patients, including ALP, iPTH, serum calcium, and serum potassium. A personalized visualized model to predict ≥ grade II complications was thereafter developed and validated by the training and validation cohorts, which exhibited strong discriminatory capability as well as excellent calibration performance. This model helps identify high-risk patients of ≥ grade II complications preoperatively, thereby guiding effective perioperative decision-making to reduce the risk of complications and improve surgical outcomes.

With advancements in perioperative management concepts, balancing radical lesion resection and complication prevention has become a core challenge in improving outcomes for SHPT patients. Previous studies predominantly focused on specific endpoints, with limited systematic analysis of overall postoperative complications, hindering cross-trial comparisons across centers. Our analysis of 788 patients revealed postoperative complications in 546 cases (69.3%), including 369 cases (46.8%) of Clavien-Dindo ≥ II grade complications. The high incidence aligns with the pathological features of metabolic derangements in SHPT patients. SHPT patients often present with calcium-phosphate imbalance, abnormal bone metabolism, and cardiovascular calcification, leading to significantly increased risks of postoperative hypocalcemia, arrhythmia, and other complications (15).

In this study, ALP levels were identified as a preoperative risk factor of ≥ grade II complications after TPTX (OR = 0.319, *P* < 0.001). As a key biomarker of bone formation, ALP directly reflects the metabolic activity of osteoblasts (16). Multiple studies have reported that elevated ALP levels indicate active bone remodeling, which increases the risk of postoperative hypocalcemia after TPTX (17–19). Specifically, a study by Ho et al. pointed out that elevated preoperative serum ALP levels are an independent risk factor for severe hypocalcemia caused by hungry bone syndrome (HBS) after parathyroidectomy (18). Tan’s study further confirmed that preoperative ALP levels can serve as a predictive indicator for early postoperative hypocalcemia, and it is recommended that patients with high preoperative ALP levels receive empirical intravenous calcium supplementation postoperatively and have their serum calcium closely monitored to reduce the occurrence of early postoperative hypocalcemia complications (19). Other scholars have found that preoperative serum ALP concentrations can also effectively predict the severity and duration of postoperative hypocalcemia (17). Once hypocalcemia occurs, it can further trigger severe complications such as arrhythmia and heart failure by inducing QT interval prolongation and myocardial electrophysiological disturbances (20). Thus, ALP may indirectly increase the occurrence of adverse cardiovascular events by increasing the incidence of postoperative hypocalcemia.Notably, the clinical significance of ALP is not limited to the field of bone metabolism. A study by Block et al. showed that serum ALP levels are closely associated with the severity of vascular calcification, and the elevation is significantly correlated with increased cardiovascular mortality in patients (21). Recent studies have also found an association between ALP and low-grade inflammatory states, and Fu’s study confirmed that patients with high preoperative ALP levels have a significantly increased risk of postoperative complications such as incision infection and pulmonary infection (22). Therefore, for patients undergoing TPTX, high preoperative ALP levels may not only significantly increase the risk of postoperative hypocalcemia but also potentially raise the risk of cardiovascular complications and infection-related complications.

As a key biomarker for assessing SHPT severity, iPTH was identified as a preoperative risk factor of ≥ grade II complications after TPTX (OR = 0.474, *P* = 0.009). Patients with higher preoperative iPTH levels may indicate more severe glandular hyperplasia or the presence of ectopic parathyroid tissue.According to the study by Sagiv et al., patients with higher preoperative iPTH levels may require more extensive surgical exploration or more complex resection procedures, thereby indirectly increasing the risk of recurrent laryngeal nerve and superior laryngeal nerve injury (23). In addition, multiple studies have clearly shown a positive correlation between preoperative iPTH levels and the risk of postoperative hypocalcemia (24–26). Our previous study also supports this conclusion, namely that high preoperative iPTH levels are a risk factor of postoperative hypocalcemia in TPTX patients (27). Sun et al. further confirmed this and suggested that high-risk patients with significantly elevated preoperative iPTH levels (especially >1000 pg/ml) should be highlighted and individualized monitoring protocols developed (25). Moreover, high iPTH levels may increase the risk of infection by interfering with immune cell function. For example, Li’s study showed that SHPT patients with higher iPTH levels had a significantly higher risk of infection-related mortality than those with lower levels (28), suggesting that patients with higher preoperative iPTH levels may have an increased risk of infection-related complications after TPTX. In conclusion, high preoperative iPTH levels may increase the risk of early postoperative complications in TPTX patients through multiple pathways, such as increasing surgical difficulty, raising the risk of postoperative hypocalcemia, and interfering with immune function, indicating that targeted monitoring and intervention measures for patients with high iPTH are needed in clinical practice.

Serum calcium is another independent risk factor of ≥ grade II complications after TPTX identified by our study. Most existing literature indicates that preoperative hypocalcemia is an independent risk factor of post-PTX hypocalcemia (27, 29, 30). In Habas’ study, it was mentioned that preoperative hypocalcemia in CKD patients reflects long-term insufficient calcium intake or absorption, resulting in reduced skeletal calcium reserves. Postoperative PTH suppression leads to enhanced osteoblastic activity, increasing the risk of hypocalcemia (29). Our previous study also demonstrated that preoperative serum calcium level is a risk factor of hypocalcemia after parathyroidectomy, and we recommend that patients with hypocalcemia should receive appropriate calcium supplementation therapy before PTX to mitigate postoperative complications of hypocalcemia (27). Additionally, Tang’s study stated that preoperative hypocalcemia significantly increases the risk of postoperative arrhythmia due to reduced extracellular calcium ion concentration prolonging myocardial action potential duration (31). Furthermore, as calcium ions are central mediators of myocardial excitation-contraction coupling (29), their depletion may further elevate the risk of postoperative heart failure. Therefore, the preoperative hypocalcemic state may increase the risk of postoperative hypocalcemia and indirectly elevate the occurrence risks of complications such as arrhythmia and heart failure.

Besides, preoperative serum potassium is independently associated with an increased risk of ≥ grade II complications after TPTX (OR = 2.500, *P* < 0.001). Our previous study has clearly identified preoperative serum potassium level as a risk factor of hyperkalemia after TPTX (32). Li’s study also confirmed it, proposing that each 1 mmol/L increase in preoperative potassium level significantly elevates the risk of postoperative hyperkalemia (33). Furthermore, a study involving 5, 959 patients demonstrated that abnormal preoperative serum potassium levels correlate significantly with higher postoperative cardiovascular complication rates in non-cardiac surgeries (34), suggesting that SHPT patients with preoperative potassium abnormalities may face elevated risks of arrhythmia and heart failure post-TPTX. Moreover, hyperkalemia directly induces arrhythmia by altering myocardial electrophysiological properties (35). Therefore, preoperative serum potassium level significantly increases the risk of postoperative hyperkalemia, and it may impact cardiovascular events after TPTX through dual mechanisms. It directly increases cardiovascular risks and indirectly exacerbates myocardial damage through hyperkalemia’s direct toxic effects.

Our study had some limitations. First, the analysis was based on single-center data, necessitating validation across multiple centers to confirm generalizability. Second, although the continuous monitoring of serum potassium, serum calcium, and other biomarkers provided detailed data for model construction, the lack of established cutoff values may limit its application in diverse clinical settings. Third, potential predictors such as preoperative bone turnover markers and novel biomarkers like fibroblast growth factor-23 (FGF-23) were not incorporated, which may hinder the model’s performance.

## 5 Conclusion

This study identified ALP, iPTH, serum calcium, and serum potassium as independent risk factors of Clavien-Dindo ≥II grade complications after TPTX in SHPT patients. Based on these four risk factors, a well-performing nomogram risk prediction model was constructed and validated. It provides clinicians with a reliable tool to promptly identify high-risk patients and promote targeted intervention measures to improve surgical outcomes.

## Data Availability

The raw data supporting the conclusions of this article will be made available by the authors, without undue reservation.
